# Utility-Oriented Placement of Actuator Nodes with a Collaborative Serving Scheme for Facilitated Business and Working Environments

**DOI:** 10.1155/2014/835260

**Published:** 2014-07-02

**Authors:** Chi-Un Lei, Woon Kian Chong, Ka Lok Man

**Affiliations:** ^1^Department of Electrical and Electronic Engineering, The University of Hong Kong, Hong Kong Island, Hong Kong; ^2^International Business School Suzhou, Xi'an Jiaotong-Liverpool University, Suzhou 215123, China; ^3^Xi'an Jiaotong-Liverpool University, Suzhou 215123, China; ^4^Yonsei University, Seoul 120-749, Republic of Korea

## Abstract

Places to be served by cyber-physical systems (CPS) are usually distributed unevenly over the area. Thus, different areas usually have different importance and values of serving. In other words, serving power can be excessive or insufficient in practice. Therefore, actuator nodes (ANs) in CPS should be focused on serving around points of interest (POIs) with considerations of “service utility.” In this paper, a utility-oriented AN placement framework with a collaborative serving scheme is proposed. Through spreading serving duties among correctly located ANs, deployment cost can be reduced, utility of ANs can be fully utilized, and the system longevity can be improved. The problem has been converted into a binary integer linear programming optimization problem. Service fading, 3D placements, multiscenario placements, and fault-tolerant placements have been modeled in the framework. An imitated example of a CPS deployment in a smart laboratory has been used for evaluations.

## 1. Introduction

Cyber-physical systems (CPS) are systems that contain numerous distributed, linked, and autonomously operated sensor nodes (SNs) and actuator nodes (ANs) [1]. Generally, CPS are used to gather adequate information about the physical environment, manipulate cyber/physical/information quantities, and, eventually, provide useful and prompt services for people [2–6]. In order to provide services, ANs interact with the physical environment by means of transducers and actuators.

In order to ensure coverage and credibility of the service, as well as minimizing resources consumed by nodes in CPS, nodes should be placed in correct positions through planning. Comparing to SNs, ANs are usually equipped with better capabilities of control, computation, and action as well as larger battery capacity. In addition, requirements and physical interaction of sensing and serving are different. Therefore, existing placement algorithms for SNs [7] are inapplicable to be directly adopted for ANs.

Placement of actor nodes (e.g., mobile robots and unmanned aerial vehicles) and mobile ANs has been explored in the literature [8–10]. Since these nodes can travel to the site of the event without obstructions, existing algorithms do not consider the uncertainty in the ability of actuators to deliver adequate service due to disturbance or fading in harsh environments. However, ordinary, small-size, and low-cost ANs are usually located in fixed positions and have different behaviors of motions and physical interactions. Therefore, algorithms for placement of actor nodes are also inapplicable for placements of ANs. Thus, specific placement frameworks for placement of ANs are needed.

Meanwhile, service coverage, system connectivity, and system longevity are typical metrics for node placement problems [7]. However, we believe that ANs in CPS should be focused on achieving a reliable and accurate serving around points of interest (POIs) with considerations of a “service utility” metric, because of the following.

- Places/objects to be concerned/served (e.g., targets in the battlefield) or crucial occurrences of an event are usually distributed unevenly over the area. Thus different areas usually have different importance and values of serving [11].
- Same type of ANs are often used in an application, but serving environments and requirements of each POI can be versatile and different [12]. As a result, serving power can be excessive or insufficient in practice. Thus, cost-effective approaches should be designed to spread serving duties among ANs.
- Balanced serving workload of ANs can prevent overloading of particular ANs beyond their limit output performance. This can avoid aging/deteriorating of equipment on ANs and, thus, improve the system longevity.

These concerns are significant yet have not been explored thoughtfully by researchers. However, in the algorithm, partial serving of POIs and utilization of redundant residual utility in ANs have not been taken into account. It is undesirable due to setup and operation cost of unnecessary ANs. Thus, it is worthwhile to develop a CPS design framework that can fully utilize the capability of ANs via a collaborative serving scheme. However, this issue does not seem to have been explored in great detail by researchers; yet, it is significant not only to many indoor (business) environments, including offices, hospitals, laboratories, schools, business facilities, and factory workshops.

Based on the preliminary study [13], a relaxed utility-oriented AN placement framework is proposed in this paper. In the framework, utility of ANs is fully utilized and the fidelity of providing service to POIs is improved. To summarize, contributions of the framework are as follows.

1. The utility-oriented placement problem has been converted into a binary integer linear programming problem. In addition, a recursive framework through the refinement of the search space has been proposed for finding a more suitable configuration.
2. Serving requirements of POIs, serving capabilities of ANs, features of delivered service, and status of serving activities have been modeled in a unified framework through a generic description. In particular, geographical attributes and obstacles can be modeled as service fading, for a more realistic modeling for deployment.
3. The proposed framework can be applied to placements for fault-tolerant serving, such that POIs are still partially served even if some ANs are damaged.
4. The proposed framework can be used for multiscenario placements, such that the system can change its serving characteristics according to the dynamic nature of the environment, without relocating ANs or consuming unnecessary energy for actions.
5. The proposed framework can be used for placements in three-dimensional (3D) fields, such that ANs can be placed on tall objects (e.g., buildings or trees) or for airborne/underwater serving.
6. An imitated example of a CPS deployment in a smart laboratory has been used for evaluations of the proposed framework.

The paper is organized as follows. Search space and derived objective function as well as constraints for the single-step placement are presented in Section 2. Generalizations for recursive placement are shown in Section 3, respectively. Finally, the performance of the framework is evaluated by examples in Section 4.

## 2. Single-Step Placement of Actuator Nodes

The objective of the framework is to pick a set of ANs and assign them to serve a set of POIs. In other words, the framework has to determine the location and service coverage radius of deployed ANs and distribution of serving workload among ANs, with a minimized total cost for setup and operation. Here, we assume that ANs can serve different POIs with different amount of utility at the same time.

The placement problem is formulated as a binary integer linear programming (BILP) problem with a cost objective function and constraints that models different concerns in the deployment of ANs. For ease of explanations, the single-step optimization framework is first described in this section. Then, the recursive framework with a refined search space is described in Section 3.

### 2.1. Search Space in the Optimization

In the framework, *I* AN candidates can be selected to serve *J* POIs. For ease of explanations, *A*_*i*_ denotes the *i*th candidate of AN and *P*_*j*_ denotes the *j*th POI, where *i* = 1,2,…, *I* and *j* = 1,2,…, *J*. The Cartesian coordinates of *A*_*i*_ and *P*_*j*_ in 2D fields are {*x*_*A*_*i*__, *y*_*A*_*i*__} and {*x*_*P*_*j*__, *y*_*P*_*j*__}, respectively. In 3D fields, the Cartesian coordinates of *A*_*i*_ and *P*_*j*_ are {*x*_*A*_*i*__, *y*_*A*_*i*__, *z*_*A*_*i*__} and {*x*_*P*_*i*__, *y*_*P*_*i*__, *z*_*P*_*i*__}, respectively.

The placement problem without mesh discretization is an integer nonlinear programming problem. To simplify computations, the infinite search space of AN locations is replaced by a search space with preselected finite elements. All AN candidates are located at the grid points, in which the grid size depends upon the accuracy of placement desired. Since the search space grows exponentially with the increase in the number of points, irrelevant candidates are neglected in the optimization problem. This is further explained in Section 2.3.

Through the discretization, the problem becomes a BILP problem. Thus, branch and bound (BB) algorithm can be used to decompose the BILP problem into linear programming (LP) problems. Optimality of the BILP solution in a finite search space is guaranteed by combining the BB framework and the simplex algorithm in LP problems. However, BILP problems are NP-complete [14, 15]. Therefore, only suboptimal solutions can be efficiently obtained via heuristic methods, such as tabu search and simulated annealing. However, it is out of the scope of this paper.

### 2.2. Cost Objective Function

The objective of the framework is to design a CPS that can serve all POIs and minimize total cost for setup and operations. The cost can be described by the following expression:

(1) $$\begin{array}{c} \min\overset{I}{\underset{i=1}{\sum }}E\times Y_{i}+r_{i}, \end{array}$$

where *Y*_*i*_ is the binary selection parameter of *A*_*i*_; that is, *Y*_*i*_ = 1 indicates candidate *A*_*i*_ is selected in the placement. Furthermore, *E* is the setup cost of an AN and *r*_*i*_ is the service coverage radius of *A*_*i*_. Generally *E* : = 100, such that all POIs are served with the minimum number of ANs. Meanwhile, {*r*_*i*_} should be minimized because ANs with a smaller coverage radius can use less power to serve, which leads to a higher energy efficiency and longevity of ANs and the system. Coverage radius can be adjusted through control units and actuators on ANs.

In some situations, for example, early design stages, we only need to find out the minimum number of required ANs for serving (*I*_min⁡_), while locations and serving coverage of ANs are unnecessary. In this case, expression (1) can be replaced by a simplified objective function for a quick cost analysis:

(2) $$\begin{array}{c} \min\overset{I}{\underset{i=1}{\sum }}Y_{i}. \end{array}$$

In the second step, specifications of ANs can then be optimized for the best system performance and largest system longevity by the following objective function:

(3) $$\begin{array}{c} \min\overset{I}{\underset{i=1}{\sum }}r_{i}\;\text{with}\;\;\overset{I}{\underset{i=1}{\sum }}Y_{i}=I_{\min}. \end{array}$$

### 2.3. Constraints on the Coverage of Services

In the framework, inspired by Elfes model [16], a relaxed disc service coverage zone centered at *A*_*i*_ is adopted in the framework. In the framework, *P*_*j*_ can be served by *A*_*i*_ if it is within the coverage of *A*_*i*_, while the serving efficiency depends on the distance between *P*_*j*_ and *A*_*i*_ (further discussions about service fading are given in Section 2.4). This restriction can be described by the following constraint:

(4) $$\begin{array}{c} r_{i}\geq d_{i,j}\times X_{i,j}, \end{array}$$

where *X*_*i*,*j*_ is the binary connection parameter between *A*_*i*_ and *P*_*j*_; that is, *X*_*i*,*j*_ = 1 indicates *P*_*j*_ is served by *A*_*i*_. *d*_*i*,*j*_ is the Euclidean distance between *A*_*i*_ and *P*_*j*_. In 2D fields, $d_{i,j}=\sqrt{{\left(x_{A_{i}}-x_{P_{j}}\right)}^{2}+{\left(y_{A_{i}}-y_{P_{j}}\right)}^{2}}$, while in 3D fields, $d_{i,j}=\sqrt{{\left(x_{A_{i}}-x_{P_{j}}\right)}^{2}+{\left(y_{A_{i}}-y_{P_{j}}\right)}^{2}+{\left(z_{A_{i}}-z_{P_{j}}\right)}^{2}}$.

In practice, ANs have a limited service coverage radius, which are based on the capability and power of circuits and devices on ANs. For simplicity, maximum service coverage radius of all ANs is *r*_max⁡_. Furthermore, if *A*_*i*_ is not selected, *r*_*i*_ : = 0. These requirements can be modeled as

(5) $$\begin{array}{c} 0\leq r_{i}\leq r_{\max}\times Y_{i}. \end{array}$$

Meanwhile, if *P*_*j*_ is within the range of *A*_*i*_, then *P*_*j*_ must be assigned to *A*_*i*_. This can be modeled by the following constraint:

(6) $$\begin{array}{c} r_{i}-d_{i,j}\leq r_{\max}\times X_{i,j}. \end{array}$$

In order to reduce the optimization problem, unnecessary AN candidates are neglected. For example, it is infeasible for an AN to serve a POI which is farther than its serving distance. Therefore, if *d*_*i*,*j*_ > *r*_max⁡_ for any *i* and *j*; *X*_*i*,*j*_ : = 0 and *q*_*i*,*j*_ : = 0. Meanwhile, it is also not practical for an AN located at the same position of POIs or obstacles. Therefore, if *A*_*i*_ is located at the same position of POIs (i.e., *d*_*i*,*j*_ = 0) or obstacles, *Y*_*i*_ : = 0.

### 2.4. Constraints on Consumption and Generation of Utilities with Collaborative Serving and Service Fading

**Figure 1:**
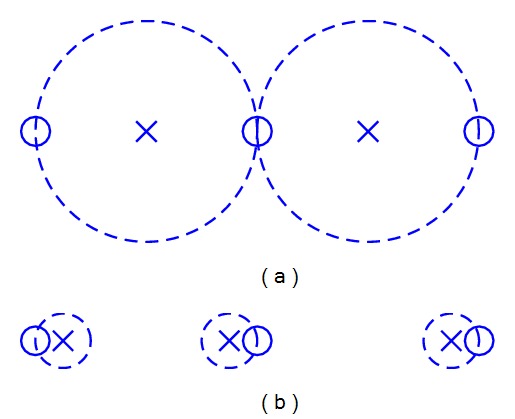
Serving of POIs: (a) if collaborative serving is allowed and (b) if collaborative serving is not allowed.

Insufficient serving power to POIs may fail to provide guaranteed services and eventually deteriorate the performance of the system. Thus, it is necessary to consider the rate/amount of utilities that can be provided by ANs (utility budget) and rate/amount of utilities that are consumed by POIs. To be specific, each POI should receive a collection of required utility *u*_*j*_ by fusing service provided by multiple ANs. In other words, *A*_*i*_ contributes part of its utility budget to serve *P*_*j*_, and *P*_*j*_ is served by contributions from several ANs. Comparing to [13], by collaborative serving, less ANs are needed to serve all POIs. An example is shown in Figure 1. Assume that after serving a POI, the residual utility of an AN is not able to serve another POI. However, if collaborative serving is allowed, the middle POI can collect enough utilities that are residual utility of its two surrounding ANs. Therefore, one AN is saved for serving. Collaborative serving can be described by the following expression:

(7) $$\begin{array}{c} C\times \left(\overset{I}{\underset{i=1}{\sum }}q_{i,j}\right)\geq u_{j}, \end{array}$$

where *u*_*j*_ is the total consumed utility of *P*_*j*_, *q*_*i*,*j*_ is the proportion of utility budget of *A*_*i*_ that contributes to *P*_*j*_, and *C* is the maximum utility budget of an AN that can serve to surrounding POIs. Meanwhile, serving capability of every AN is limited by its utility budget. Therefore, the following inequality is required in the problem:

(8) $$\begin{array}{c} \overset{J}{\underset{j=1}{\sum }}q_{i,j}\leq 1. \end{array}$$

If part of the utility budget in *A*_*i*_ is used to serve *P*_*j*_, *A*_*i*_ is selected for serving and it is responsible to serve *P*_*j*_. These can be described by the following inequalities:

(9) $$\begin{array}{c} \overset{J}{\underset{j=1}{\sum }}q_{i,j}\geq Y_{i},\\ X_{i,j}\geq q_{i,j}. \end{array}$$

In practice, the strength of service power usually decays when the service is delivered from the AN to the POI. In other words, an AN can serve several POIs that are close to the AN, but it can only serve one POI that is far from the AN. In order to describe this phenomenon, (7) can be replaced by the following constraint:

(10) $$\begin{array}{c} C\times \left(\overset{I}{\underset{i=1}{\sum }}q_{i,j}\times {\left(\frac{f_{i,j}}{d_{i,j}+g}\right)}^{2}\right)\geq u_{j}, \end{array}$$

where *f*_*i*,*j*_ is the factor for path loss of the delivered service from *A*_*i*_ to *P*_*j*_ and *g* is the factor for the operation loss of an AN. {*f*_*i*,*j*_} and *g* and {*u*_*j*_} and *C* can be determined through product specifications, theoretical estimations, or experimental measurements. In brief,

- {*u*_*j*_} depends on scenarios of serving. In particular, it depends on accuracy, reliability, relevance, timelessness, and usability of service. It also depends on whether the service is periodic or event-driven. In general, {*u*_*j*_} should be higher for critical POIs, such that allocated AN(s) will pay more attention to these POIs. For example, the POI may require a larger {*u*_*j*_} if there is a higher possibility of making a significant damage, due to the severity of crashes and shocks.
- {*f*_*i*,*j*_} depends on features and decay rate of the service and the ambient environment as well as the disturbance between ANs and POIs (e.g., trees and walls). In general, ANs can serve targets that lie in their line of sight; therefore, {*f*_*i*,*j*_} should be larger; on the other hand, obstacles may make the service unreachable; therefore, {*f*_*i*,*j*_} should be smaller.
- *C* and *g* depend on the battery capacity, power dissipation and characteristics of actuators, onboard circuitry, and peripherals on ANs.

The proposed generic constraint can be further generalized by including specific considerations [17, 18], such as battery models with energy harvesting capabilities [17]. However, the discussion of utility generalizations is beyond the scope of the paper.

### 2.5. Constraints for Fault-Tolerant Serving and Serving with Limited Recipients per AN

Generally, every POI is served by the minimum number of ANs. However, due to the impact from environment hazards and physical shock, ANs may be damaged or even failed to operate after deployment. In these dynamic situations, the system is expected to tolerate failures of some ANs and communication link as well as guaranteeing a proportion of serving ability to POIs. Introducing adequate redundant ANs can surely ensure the connectivity. However, it also requires more installed ANs, which can be undesirable due to the cost. In our framework, fault-tolerant serving can be done by ensuring each POI is served by at least *K* AN(s), such that at least POIs are partially served by ANs if minorities of ANs are broken. This design restriction can be described by the following connection constraint, for any *P*_*j*_:

(11) $$\begin{array}{c} \overset{I}{\underset{i=0}{\sum }}X_{i,j}\geq K. \end{array}$$

In the framework, we assume that ANs can serve unlimited POIs at the same time. However, sometimes this assumption becomes impractical because of the hardware capabilities of ANs. In these situations, we need to ensure that each AN can only serve at most *L* POIs. This design restriction can be described by the following connection constraint, for any *A*_*i*_:

(12) $$\begin{array}{c} \overset{J}{\underset{j=0}{\sum }}X_{i,j}\leq L. \end{array}$$

### 2.6. Multiscenario Placement through a Relaxation of Constraints

In the proposed framework, ANs are placed in fixed locations and serve POIs according to a single scenario. However, serving patterns and requirements can be changed according to the pattern of events as well as application-level interests. For example, requirements of air ventilation during daytime and night-time can be different. In such circumstances, a fixed arrangement is not capable to consider dynamic changes for a better delivery of services. Therefore, in order to improve the feasibility of meeting timeliness requirements, ANs' service should be rescheduled without node relocation. Through the proposed generalization, the framework can place ANs with considering multiple serving scenarios.

Assume that there are *K* serving scenarios, in order to distribute serving duties to ANs for each scenario correctly, {*X*_*i*,*j*_} and {*q*_*i*,*j*_} in (7)–(11) are replaced by {*X*_*i*,*j*,*k*_} and {*q*_*i*,*j*,*k*_}, where *X*_*i*,*j*,*k*_ is the binary connection parameter *X*_*i*,*j*_ and *q*_*i*,*j*,*k*_ is the proportion parameter *q*_*i*,*j*_, for the *k*th scenario, respectively, for *k* = 1,…, *K*. Meanwhile, in order to determine the coverage radius of ANs, {*X*_*i*,*j*_} are used as usual for (4)–(6). Furthermore, *A*_*i*_ is selected even if *A*_*i*_ is only selected in one of the scenarios; that is, *X*_*i*,*j*_ = 1 if *X*_*i*,*j*,*k*_ = 1 for any *k*, and *X*_*i*,*j*_ = 0 if *X*_*i*,*j*,*k*_ = 0 for all *k*. Therefore, determination of {*X*_*i*,*j*_} is enforced by the following linear inequality constraints, such that the generalized problem can still be solved by a BILP optimization process:

(13) $$\begin{array}{c} X_{i,j}\geq X_{i,j,k},\;\text{for}\;\;k=1,\ldots ,K, \end{array}$$

(14) $$\begin{array}{c} X_{i,j}\leq \overset{K}{\underset{k=1}{\sum }}X_{i,j,k}. \end{array}$$

## 3. Recursive Placement via a Refined Search Space

The single-step placement framework described in Section 2 can be generalized into a recursive framework for a better placement. The algorithm pursues a divide-and-conquer strategy to split the overall placement problem into a series of placement problems with smaller size (in terms of area for placement), such that the single-step framework can be executed recursively.

We assume that if a set of ANs {*A*_selected_} is obtained through the single-step framework, a better placement can be obtained from the neighbourhood of {*A*_selected_}. Here, neighbors of {*A*_selected_} are located within [*x*_*A*_selected__ ± (Δ*x*_*A*_/2), *y*_*A*_selected__ ± (Δ*y*_*A*_/2)], where Δ*x*_*A*_ and Δ*y*_*A*_ are the resolution of the original search space. In the recursive framework, based on the location of {*A*_selected_}, a new set of candidates can be generated. Meanwhile, through the new set of AN candidates, a new set of ANs with a better placement can be found. Thus, the placement can be repeated recursively until the replacement of ANs cannot further reduce the operation cost in terms of average {*r*_*i*_}, which can be simplified as ||{*r*_*i*_}_previous_−{*r*_*i*_}_current_||_2_/||{*r*_*i*_}_previous_||_2_ ≤ *ε*, where *ε* is the predefined tolerance. In that case, selected AN candidates and their positions are declared as the final answer and the algorithm terminates. In summary, pseudocodes of the recursive placement framework are shown in Algorithm 1.

## 4. Performance Evaluation of the Framework

In this section, examples are used to show the performance of the framework. Computations run in an optimization solver Lingo 11 [19] on a 6 GB-RAM Intel i7 3.4 GHz PC.

### 4.1. Placement in a 2D Laboratory Environment

**Figure 2:**
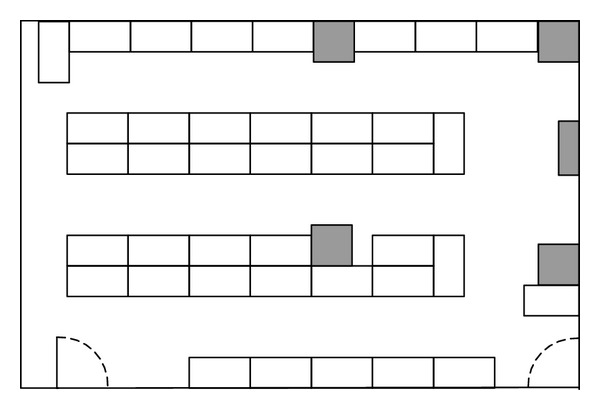
The floor-plan of the laboratory in the 2D field example. The grey squares and white rectangles denote wall structures and laboratory benches, respectively.

**Figure 3:**
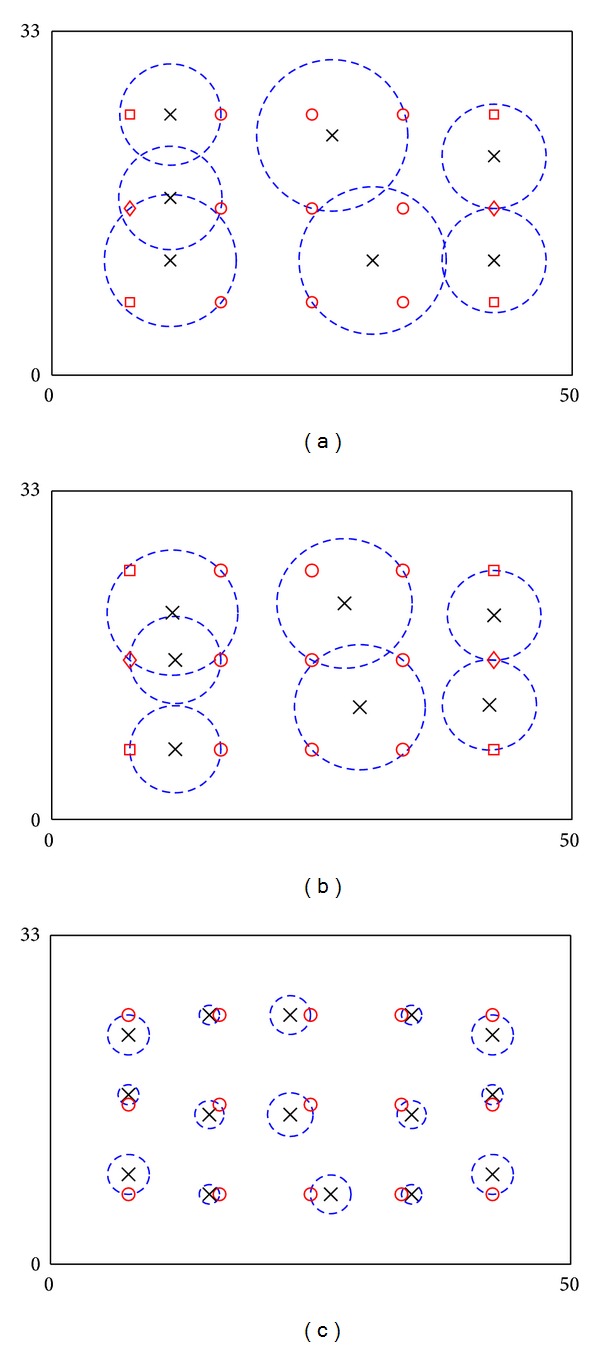
Locations of POIs and ANs in the 2D field example via the proposed framework: (a) optimization with *r*_max⁡_ = 10 m, (b) optimization with *r*_max⁡_ = 10 m after five iterations through recursive placement, and (c) optimization with *r*_max⁡_ = 1 m. Crosses and dash circles denote ANs and service coverage of ANs. Circles, squares, and diamonds denote POIs with 25, 40, and 55 consumed utilities, respectively.

The performance of the system is analysed via a simulated example of a school laboratory with dimensions 50 m × 33 m [20–22], as shown in Figure 2. In order to monitor and regulate ventilations and thermal comfort of the laboratory, a CPS with sensors and actuators (i.e., electrical fans) is installed. A total 15 points of interest (POIs) and their consumed utilities are assigned according to positions of vents and pattern of activities in the laboratory, as shown in Figure 3. In the example, service fading is excluded and single-step placement is used. Before the optimization, 100 candidates of ANs, which are located in areas bounded by outmost ANs, are generated. For every candidate, utility budget of an AN (*C*) is assumed to be 75 and *r*_max⁡_ = 10 m. After computations, the framework suggests that seven ANs with average {*r*_*i*_} = 5.785 m are required to provide adequate requested services to all POIs. Results are shown in Figure 3(a). Figure shows that some ANs serve one critical POI and partially serve one noncritical POI, while others serve at most three noncritical POIs. Meanwhile, in some cases, one POI is served by two ANs. The problem contains 1036 variables (including 468 binary variables) and 4817 constraints, and the algorithm needs 30 minutes to obtain the optimal solution. Results with *r*_max⁡_ = 1 m are also shown in Figure 3(c). Figure 3(c) shows that if *r*_max⁡_ is small, ANs work independently without sharing their duties.

### 4.2. Placement via a Recursive Framework

The recursive framework is then applied to the example in Section 4.1. In the example, 100 AN candidates have been used for optimization in the first iteration. 25 (5 × 5) candidates have been generated for each AN. Since seven ANs are needed for placement, totally 175 candidates have been used for optimization after the first iteration. In every iteration, the algorithm terminates automatically after one hour if it cannot obtain an optimal solution. The obtained solution is then used to refine the search space for the next iteration. After five iterations, average coverage radius of ANs has been reduced by >8.98%. And the placement is shown in Figure 3(b). More details of results are shown in Table 1. Results show that recursive framework can improve the performance of ANs, with the expense of computation time.

### 4.3. Comparison with Other Strategies for Placements

**Figure 4:**
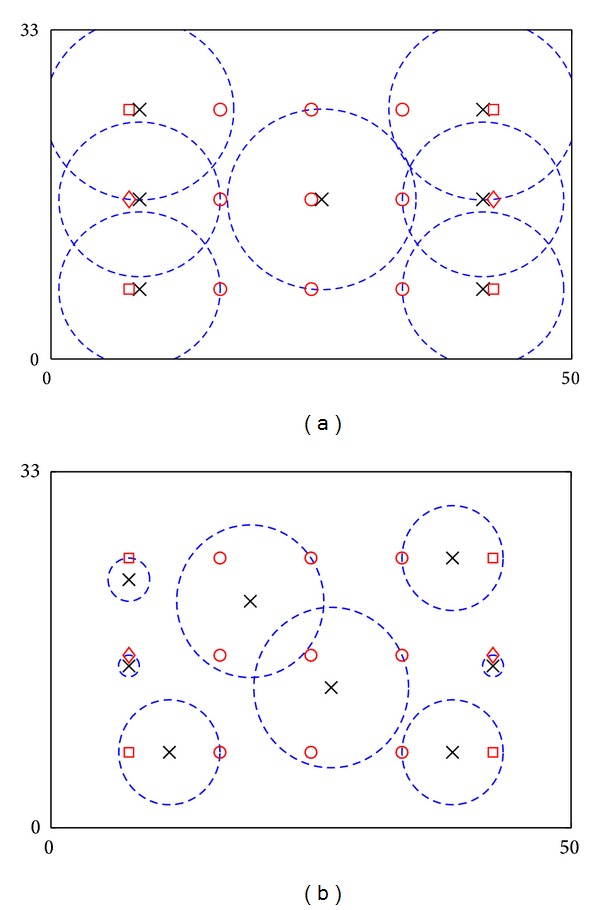
Locations of POIs and ANs in the 2D field example: (a) placement using Strategy I and (b) placement using Strategy II. Crosses and dash circles denote ANs and service coverage of ANs. Circles, squares and diamonds denote POIs with 25, 40, and 55 consumed utilities, respectively.

Results of the proposed framework are compared with the following three strategies: (i) ANs are placed near the location of POIs one by one, with serving POIs with the highest utility consumption first and with utilization of residual utilities (Greedy approach, Strategy I), (ii) ANs are placed with considerations of geographical distribution of POIs only (Strategy II), and (iii) ANs are randomly placed with utilization of residual utilities (Strategy III). The example in Section 4.1 is used for evaluation. Results of the first two strategies are shown in Figure 4. Figures show that if Strategy I is used, seven ANs with average {*r*_*i*_} = 8.309 m are needed to serve all POIs. Meanwhile, if Strategy II is used, the system serves all POIs by eight ANs with average {*r*_*i*_} = 3.891 m. Furthermore, Strategy III has been tested ten times. Averagely, the system can serve all POIs by seven ANs with average {*r*_*i*_} = 11.546 m. Therefore, the proposed framework outperforms the other three strategies in terms of the number of ANs (i.e., system cost) or average {*r*_*i*_} (i.e., operation efficiency and system longevity). This is because the proposed framework considers geographic distribution of POIs and utilization of residual utilities for placements at the same time. These considerations become even more significant if the number of ANs is limited. These situations are typical because ANs are usually expensive to deploy.

### 4.4. Placements with Different Configurations

Example in Section 4.1 is used for investigating the influence of various configurations. Results are shown in Table 2. From examples, we can arrive at a few conclusions about the result of placements as well as the efficiency of the framework.

- In general, the system requires fewer ANs as the utility budget (*C*) and maximum service coverage radius (*r*_max⁡_) of ANs increase. For example, only one AN is needed to serve the whole room if the actuator has an extremely large *C* and *r*_max⁡_. However, this assumption is usually not practical. Therefore, *C* and *r*_max⁡_ should be configured carefully.
- The space complexity of the optimization mainly depends on the number of POIs and number of AN candidates, while the time complexity mainly depends on the utility budget (*C*) and maximum service coverage radius (*r*_max⁡_). This is because there are usually more combinations of duty distributions between ANs for a larger *C* or *r*_max⁡_.
- When the utility consumption is dominated by a few POIs or the utility budget of ANs is small, ANs tend to work independently without sharing job duty. On the other hand, when the utility consumption is evenly distributed, there is a higher possibility for ANs to share their workload. In this situation, the proposed framework can achieve a better performance.
- If each AN can only serve at most two POIs due to limitations of hardware on ANs, through including constraint (12) for every ANs, the framework suggests that nine ANs with average {*r*_*i*_} = 4.238 m are needed to serve all POIs.

### 4.5. Impact of Fading on the Delivery of Services

Example in Section 4.1 with different *f* and *g* = 0.01 is used for investigating the influence of service fading. Results are shown in Table 3. Results show that the number of required ANs increases as *f* decreases. It is because for a smaller *f* (i.e. a larger service power fading), POIs that are far from ANs consume more utility. In some situations, POIs that are too far from ANs cannot be served. As a result, additional ANs are added to serve these POIs. For example, if *f* = 1.5 m, workload cannot be shared among ANs; therefore, every POI is individually served by a nearby AN.

**Figure 5:**
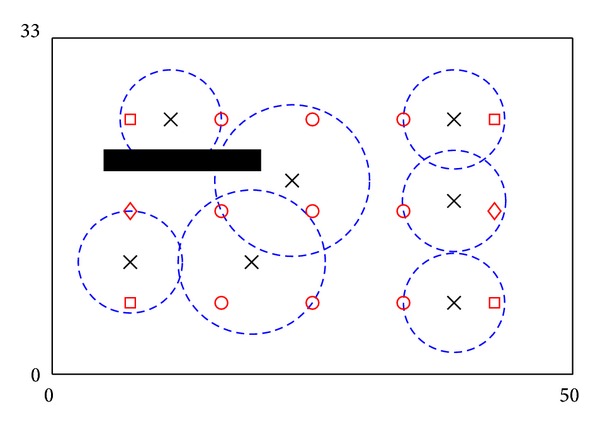
Locations of POIs and ANs in the 2D field example with the inclusion of an obstacle. Crosses and dash circles denote ANs and service coverage of ANs. Circles, squares, and diamonds denote POIs with 25, 40, and 55 consumed utilities, respectively. The black block denotes the obstacle.

In the second example, an obstacle has been included, as shown in Figure 5. In the example, we assume the obstacle can effectively block the service and ANs can only serve POIs that lie in its line of sight. In order to model the obstacle, if the line of sight between *A*_*i*_ and *P*_*j*_ is blocked (i.e., the service is very difficult to be delivered from *A*_*i*_ to *P*_*j*_), *f*_*i*,*j*_ : = 0.05 m; otherwise, *f* : = 0.55 m. Furthermore, some AN candidates are neglected since the obstacles have occupied spaces of some AN candidates. If the original placement is used, ANs are not able to serve some POIs. The new placement in Figure 5 shows that the system can still serve all POIs by placing ANs with similar configurations in different positions.

### 4.6. Multiscenario Placement

**Figure 6:**
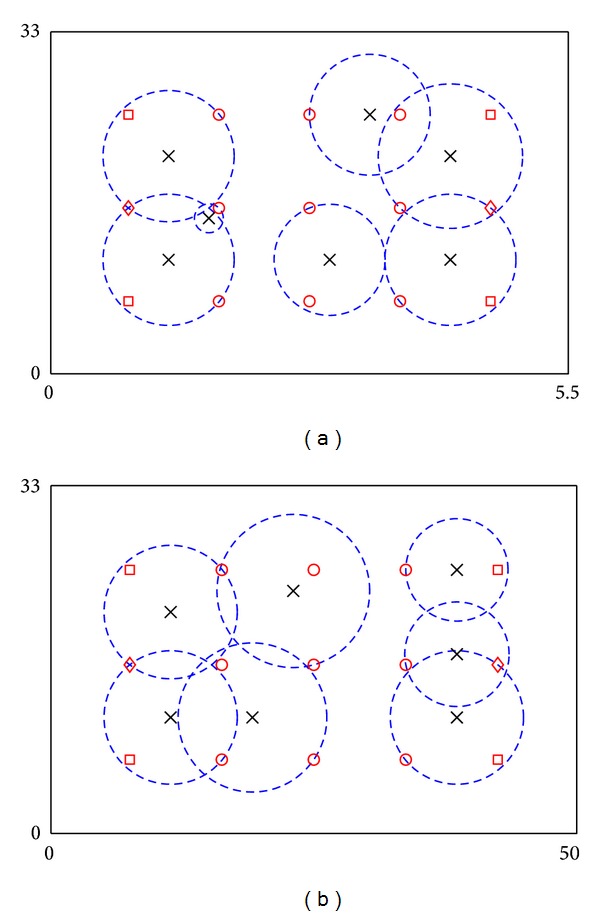
Locations of POIs and ANs in the 2D field example: (a) placement with Scenario B and (b) multiscenario placement with Scenarios A and B. Crosses and dash circles denote ANs and service coverage of ANs. Circles, squares, and diamonds denote POIs with 25, 40, and 55 consumed utilities in Scenario A and 35, 25, and 25 consumed utilities in Scenario B, respectively.

In this example, the system has to ensure the delivery of services in two scenarios: (i) the distribution of utility consumption in Section 4.1 is used as serving for office hours (Scenario A), and (ii) Figure 6(a) shows the distribution of serving for nonoffice hours (Scenario B). Figure 3(a) and Figure 6(a) show the placement of ANs if only Scenario A or B is considered, respectively. Meanwhile, Figure 6(b) shows the placement if both scenarios are considered for placements. The figure shows that 14 ANs with average {*r*_*i*_} = 5.174 m are needed to serve all POIs. Therefore, the framework can be used for multiscenario placements without introducing extra ANs.

### 4.7. Fault-Tolerant Placement with a Large-Scale Example

**Figure 7:**
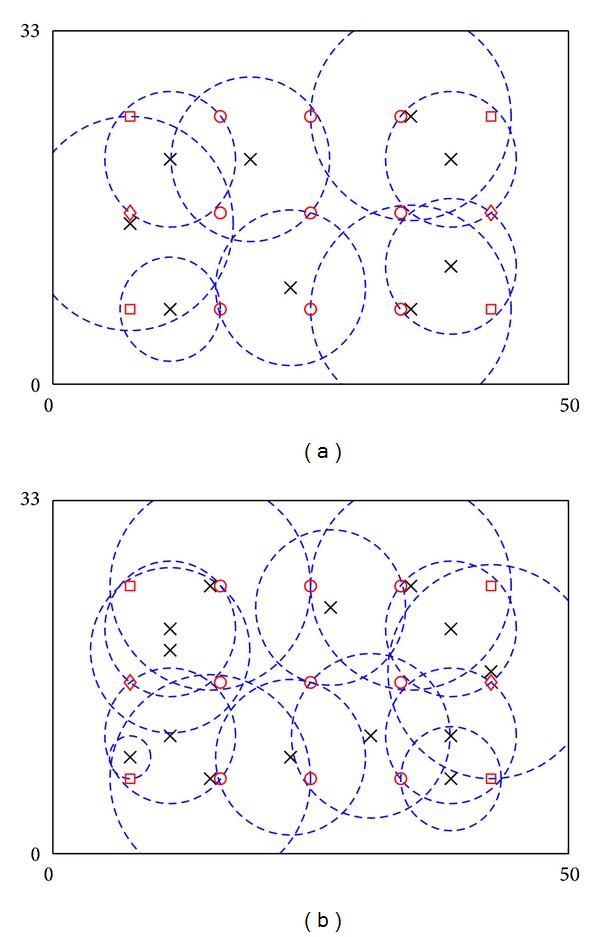
Locations of POIs and ANs in the 2D field example: (a) fault-tolerant placement with *K* = 2 and (b) fault-tolerant placement with *K* = 3. Crosses and dash circles denote ANs and service coverage of ANs. Circles, squares, and diamonds denote POIs with 25, 40, and 55 consumed utilities, respectively.

In order to demonstrate the fault-tolerant placement, the example in Section 4.1 with fault-tolerant parameters *K* = 2 and *K* = 3 is used. Placements of ANs for these two situations are shown in Figure 7. Figures show that nine ANs with average {*r*_*i*_} = 7.5841 m are necessary if each POI is served by at least two ANs (i.e., *K* = 2). Meanwhile, 14 ANs with average {*r*_*i*_} = 7.2357 m are necessary if POIs are served by at least three ANs (i.e., *K* = 3). Figures show that in order to ensure POIs can be partially served by ANs, only a few extra ANs are needed. Therefore, the algorithm allows ANs to provide fault-tolerant service through a cost-effective approach.

**Figure 8:**
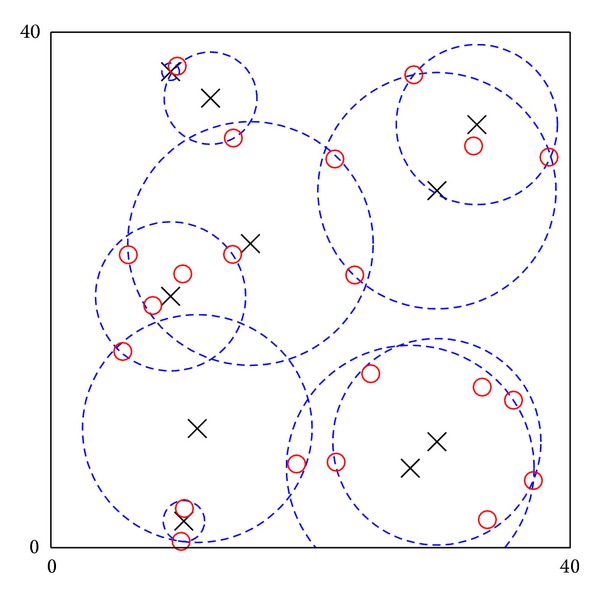
Placement of ANs in a large-scale example with *K* = 2. Crosses and dash circles denote ANs and service coverage of ANs. Circles, squares, and diamonds denote POIs with 25, 40, and 55 consumed utilities, respectively.

The second example is a large-scale example with 21 POIs. Their location and consumed utility are randomly assigned. A search space with 400 candidates has been generated for optimization. If *K* = 2, the framework requires 130 minutes to obtain the optimal solution, and the result is shown in Figure 8. The example illustrates that the proposed framework can solve large-scale placement problems.

### 4.8. Placement in a 3D Field Environment

The performance of the framework is analysed via a simulated example of an arbitrary 3D environment with dimensions 80 m × 80 m × 80 m. A total of seven POIs are distributed randomly, with a random consumed utility ranging from 23 to 32. Totally, 512 candidates of ANs are distributed evenly in the 3D field. Through the optimization, six ANs with average {*r*_*i*_} = 7.594 m are assigned to provide adequate requested services for all POIs. The framework needs 33 seconds to solve the problem. The example proves that the proposed framework can be used for placements of ANs in 3D fields.

## 5. Conclusion

An actuator node placement framework with collaborative sharing of utility has been presented. In particular, partial serving and utilization of redundant residual utility in ANs are allowed in the relaxed formulation. Service fading, 3D placements, multiscenario placements, and fault-tolerant placement have been modeled in the framework. The problem has been converted into a binary integer linear programming optimization problem, such that the optimal solution can be obtained in a discretized search space.

We believe that this research is not only confined to schools, hospitals, and factory workshops, but also to many similar business environments such as offices. These environments are important to create positive benefits to the society as well as business activities. If we are to develop smart societies, we need to look at systems that are easily deployable, noninvasive, economical, effective, and energy efficient. Our algorithm for actuator node placement shows we can satisfy all these conditions.

In the future, we will generalize the framework for duty scheduling of ANs or robots in CPS, which aims at awaking several ANs or robots to work while putting others in sleep mode, such that the system longevity can be extended. Further, to improve serving power, a smart power/battery management system will be potentially integrated into the framework.

## Figures and Tables

**Algorithm 1:**
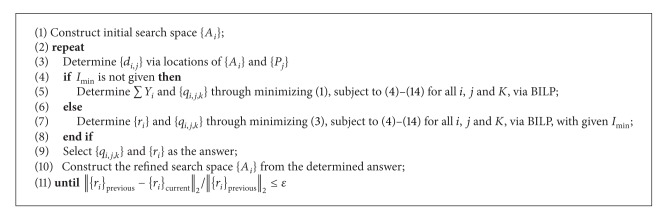
Pseudocodes of the recursive framework.

**Table 1:** Results of the example via the recursive framework.

*n*th iteration	1	2	3	4	5
Average coverage radius (m)	5.7852	5.4523	5.2755	5.2706	5.2657

**Table 2:** Results and computation complexity with respect to different problem configurations.

*C*	*r*_max⁡_ (m)	Number of required ANs	Average {*r*_*i*_} (m)	CPU time (min.)
75	10	7	5.785	30
75	5	11	2.755	0.10
75	4.5	15	0.984	0.10

75	10	7	5.845	5
75	10	7	5.845	10
75	10	7	5.785	15
75	10	7	5.785	107 (Optimal)

50	10	10	5.288	60
25	10	20	2.5248	60
1000	25	1	21.865	0.58 (Optimal)

**Table 3:** Impact of service fading on the delivery of services with different *f*.

*f* (m)	Number of required ANs	Average coverage radius (m)	Average consumed utility (Scaled)	Average consumed utility (Original)
7.5	5	8.142	99.000	74.627
6.0	6	8.123	82.500	71.778
5.5	7	5.580	70.714	56.520
5.0	8	4.463	61.875	46.358
4.0	9	4.032	55.000	71.925
1.5	15	0.984	33.000	0.208
